# Two-dimensional ECG-based cardiac arrhythmia classification using DSE-ResNet

**DOI:** 10.1038/s41598-022-18664-0

**Published:** 2022-08-25

**Authors:** Jiahao Li, Shao-peng Pang, Fangzhou Xu, Peng Ji, Shuwang Zhou, Minglei Shu

**Affiliations:** 1 School of Electrical Engineering and Automation, Qilu University of Technology (Shandong Academy of Science), Jinan, 250353 Shandong Province China; 2 School of Electronic and Information Engineering (Department of Physics), Qilu University of Technology (Shandong Academy of Science), Jinan, 250353 Shandong Province China; 3 Qilu University of Technology (Shandong Academy of Sciences), Shandong Artificial Intelligence Institute, Jinan, 250014 China

**Keywords:** Imaging techniques, Cardiology, Information technology

## Abstract

Electrocardiogram (ECG) is mostly used for the clinical diagnosis of cardiac arrhythmia due to its simplicity, non-invasiveness, and reliability. Recently, many models based on the deep neural networks have been applied to the automatic classification of cardiac arrhythmia with great success. However, most models independently extract the internal features of each lead in the 12-lead ECG during the training phase, resulting in a lack of inter-lead features. Here, we propose a general model based on the two-dimensional ECG and ResNet with detached squeeze-and-excitation modules (DSE-ResNet) to realize the automatic classification of normal rhythm and 8 cardiac arrhythmias. The original 12-lead ECG is spliced into a two-dimensional plane like a grayscale picture. DSE-ResNet is used to simultaneously extract the internal and inter-lead features of the two-dimensional ECG. Furthermore, an orthogonal experiment method is used to optimize the hyper-parameters of DSE-ResNet and a multi-model voting strategy is used to improve classification performance. Experimental results based on the test set of China Physiological Signal Challenge 2018 (CPSC2018) show that our model has average $F_1= 0.817$ for classifying normal rhythm and 8 cardiac arrhythmias. Meanwhile, compared with the state-of-art model in CPSC2018, our model achieved the best $F_1$ in 2 sub-abnormal types. This shows that the model based on the two-dimensional ECG and DSE-ResNet has advantage in detecting some cardiac arrhythmias and has the potential to be used as an auxiliary tool to help doctors perform cardiac arrhythmias analysis.

## Introduction

The ECG^1^ records the electrical signals of the human heart and is mostly used for clinical diagnosis of cardiac arrhythmias. More than 300 million ECGs are obtained worldwide every year^2^. The huge diagnostic workload leads to inefficiency and misdiagnosis of cardiac arrhythmias based on ECG. So the combination of extensive digitization of ECG data and automatic classification algorithms has attracted more and more attention.

In the early research on the automatic classification of cardiac arrhythmia, most algorithms based on machine learning are usually divided into two parts: feature engineering and classification. Specifically, researchers first manually extracted a large number of ECG features with medical meaning, such as wavelet features^3^, P-QRS-T composite features^4–6^, heart rate variability statistical feature^7^, RR-related statistical features^8,9^, higher order statistical features^10^ and morphological features^11–14^. Meanwhile, the principal component analysis^15,16^ and independent component analysis^17,18^ use mathematical methods to extract ECG features from high-dimensional space to low-dimensional space. After feature engineering, support vector machine^19–21^, self-organizing map^22^, clustering^23^ and other machine learning algorithms are used to analyze artificial features and give the prediction result. Although machine learning has broad research applications in the classification of cardiac arrhythmia, there are still some problems that need to be solved. For example, feature engineering based on subjective factors leads to the elimination of some potentially important features, which may affect the final classification performance.

In recent years, DNNs have greatly improved the technical level of speech recognition, image classification, strategy games, and medical diagnosis by virtue of their powerful feature extraction capabilities and incremental learning methods. Different from machine learning methods, DNNs can recognize patterns and learn useful features from raw input data without requiring a lot of manual rules and feature engineering, making them particularly suitable for interpreting ECG data. Some studies have been inspired to use DNNs for the automatic classification of cardiac arrhythmia based on single-lead or multi-lead ECG. For example, Ullah et al.^24^ converted single-lead ECG into the 2D spectral image, and used 2D-CNN to learn the features of the image to achieve the automatic classification of cardiac arrhythmias, their model achieved average classification accuracy of 99.11% in the MIT-BIH dataset. Hannun et al.^25^ developed a DNN to classify 12 rhythm categories based on single-lead ECG. The experiments found that the average $F_1$ score (0.837) of their DNN models exceeded the average score of cardiologists (0.780). This demonstrates that the end-to-end deep learning approach can enable identification of a wide range of cardiac arrhythmias based on single-lead. At the same time they mentioned that factors such as limited signal duration or only one lead limit the valid conclusions that can be drawn from the data. Compared with single-lead, multi-lead ECG contains more valuable information^2,26^, which is more conducive to the automatic classification of cardiac arrhythmia. Zhang et al.^2^ proposed an interpretable DNN for automatic diagnosis based on 12-lead ECG. Their experiments have demonstrated that the performance of DNN trained on single-lead ECG is lower than that produced by using all 12-lead simultaneously. Wang et al.^27^ proposed a method based on multi-scale feature extraction and 12-lead ECG cross-scale information complementation to capture the abnormal state in ECG. Their model based on this approach achieved $F_1$ score of 0.841 in the PhysioNet/CinC_2017 dataset. Chen et al.^28^ proposed a neural network that combines convolutional neural networks (CNNs), recurrent neural networks, and attention mechanisms for cardiac arrhythmias classification. Their model won the state-of-art of $F_1$ score (0.837) in CPSC2018^29^. Ribeiro et al.^30^ proposed a DNN model trained on a dataset with more than 2 million labeled exams and found that the model achieved $F_1$ score> 0.8 and specificity > 0.99, which outperformed heart disease doctor’s diagnosis. In addition, Zhao et al.^31^ fed the patient’s age and gender as auxiliary information into the DNN, and the DNN model achieved the second-ranked test result in the PhsioNet/Computing in Cardiology Challenge 2020.

These studies promote the application of deep learning in the automatic classification of cardiac arrhythmia. However, some studies on the automatic classification of cardiac arrhythmias based on single-lead of ECG suggest that only one lead may lead to DNN misclassification. This drove us to choose 12-lead rather than single-lead as experimental data. Partly based on the 12-lead DNN training process is divided into two steps, firstly train the leads one by one, then fuse the trained features of each lead, and finally get the classification result. This leaves no attention to the relationship between leads at the beginning of training. Based on these problems, we propose two-dimensional ECG and DSE-Resnet. The main contributions of this work can be summarized as follows:

- A two-dimensional method of converting multi-channel time-series signals is proposed. The original 12-lead ECG is spliced into a 2D plane like a grayscale picture, where each column represents the time-series of a single-lead, and each ’pixel’ represents a voltage value of ECG.
- A two-dimensional CNN model DSE-ResNet is proposed for processing multi-channel time series ECG signals. DSE-ResNet can learn both internal and inter-lead features during the training phase.
- A slicing rule is proposed to expand the training set.
- Orthogonal experiments are used to select hyper-parameters. In the evaluation model stage, we use ensemble learning based on a voting strategy to obtain classification performance.

## Materials and methods

### Problem definition

This paper aims to realize the automatic classification of normal rhythm and 8 cardiac arrhythmias based on the 12-lead ECG records. The input *x* of the proposed model includes 2D ECG signals and basic information about the patients, and the output is the predicted labels corresponding to the normal rhythm and 8 cardiac arrhythmias. The inputs and reference label *y* constitute the training set $X = \{(x_1, y_1), (x_2, y_2), \ldots , (x_n, y_n)\}$. The training goal of our model is to minimize the softmax cross-entropy loss function within a finite number of training epochs, where the softmax cross-entropy loss function is

1 $$\begin{aligned} \mathrm{Loss}(X) = -\frac{1}{n}\sum _{i=1}^n\log {\frac{\exp (p(x_i, y_i)}{\sum _j \exp (p(x_i, y_j))}}, \end{aligned}$$

where $p(x_i, y_i)$ and $p(x_i, y_j)$ represent the probability that the model predicts input $x_i$ to the reference label $y_i$ and the other label $y_j$, respectively.

### Two-dimensional ECG

#### Data sources

The 12-lead ECG dataset^32^ used in this paper came from CPSC2018, which was sampled at 500 Hz and collected from 11 hospitals. It has 9831 samples, where 6877 (female: 3178 and male: 3699) samples were released for training and 2954 samples were kept private for testing. Each sample contains the 12-lead ECG signals, basic information of the patient (age and gender) and the reference label, where label corresponds to 9 categories: Normal rhythm, Atrial fibrillation (AF), First-degree atrioventricular block (I-AVB), Left bundle brunch block (LBBB), Right bundle brunch block (RBBB), Premature atrial contraction (PAC), Premature ventricular contraction (PVC), ST-segment depression (STD) and ST-segment elevated (STE). More details of the data sources are shown in Table 1.

**Table 1:** Data profile.

No.	Type	Record	Time length			Training set	Small test set	Hidden test set
			Mean	Min	Max			
1	Normal	918	15.43	10	64	895	69	394
2	AF	1098	15.04	9	74	1112	79	466
3	I-AVB	704	14.27	10	54	695	45	295
4	LBBB	207	14.94	9	65	203	15	97
5	RBBB	1695	14.62	10	118	1691	124	756
6	PAC	574	19.43	9	74	546	47	250
7	PVC	653	20.92	6	144	826	44	276
8	STD	826	15.50	8	138	825	58	340
9	STE	202	17.15	10	60	216	19	80
	Total	6877	15.95	6	144	7117	500	2954

#### Two-dimensional processing

In practice of clinical medicine, cardiologists usually need a multi-lead ECG as a basis for detection of cardiac arrhythmias. For example, the ECG abnormalities of patients with PAC were usually manifested in the leads V1, II, and aVF, and the typical abnormal ECG of patients with LBBB was mainly appeared in the leads I, V1, V2, V5, V6 and aVR^33^. The detection of different cardiac arrhythmias requires the comprehensive information of 12-lead ECG, which means that both internal and inter-lead features play an important role in the classification of cardiac arrhythmia.

**Figure 1:**
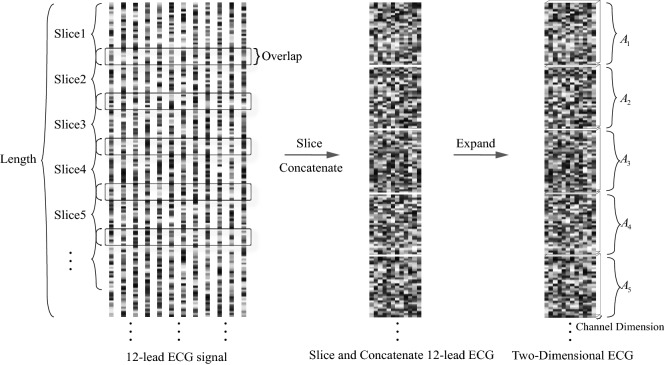
Two-dimensional and expend dimension process.

In order to extract the internal and inter-lead features of the 12-lead ECG at the same time, we perform two-dimensional processing on the 12-lead ECG. Specifically, the leads $a\in \mathbb {R}^{L\times 1}$ are spliced together to form a matrix $A\in \mathbb {R}^{ L\times 12}$, where *L* is the length of leads. As shown in Fig. 1, the original 12-lead ECG is spliced and concatenated into a two-dimensional plane like a grayscale picture, where each column represents the time series of one lead, and each ’pixel’ represents a voltage value of ECG.

#### Slicing

It can be seen from the Table 1 that the number of records in normal rhythm and 8 cardiac arrhythmias is quite different, and the length of the original 12-lead ECG is also different. In order to make full use of the data and unify the length of the 12-lead ECG, we sliced the two-dimensional ECG.

The ECG dataset of CPSC2018 contains 6877 training signals. Because the test set of CPSC2018 is not open to the public, we separated 500 sets of data from the 6877 sets of open access data as the offline small number of test set. The main role of the small number of test set is to compare the performance of the sub-model and the ensemble model. The 12-lead ECG in the remaining 6377 ECG signals were sliced and used for training. The specific steps of slicing are as follows:

1. If the length of a two-dimensional ECG *A* is $L < 8192$, the length of *A* is filled with zeros to $L= 8192$.
2. If the length of *A* satisfies $8192 \le L < 1.5\times 8192$, *A* is cut off the extra data at the tail to $L= 8192$.
3. If the length of *A* satisfies $L \ge 1.5\times 8192$, *A* is sliced into *n* pieces. The slice length is 8192, and the overlap length between slices is 4096. The number of slices is $n = \lfloor \frac{2 L}{8192} \rfloor - 1$, where $\lfloor x \rfloor$ represents the largest integer less than *x*.

It is important to note that the slice length determines the length of the 12-lead signal input into the DNN. There are multiple 0.5 times downsampling processes in DSE-ResNet. In order to facilitate dimension statistics after downsampling, we choose the length of the exponential power of 2 as the slice length. At a sampling rate of 500Hz, a slice length of 8192 represents a 12-lead signal length of approximately 16.384s. We counted the length distribution of the original samples in CPSC2018^29^. The average length of the samples is 15.95s, so we choose the closest 8192 (16.384s) as the slice length. 12.7% of the samples are more than 1.5 times the average length, and we called this part of the samples with more ECG information. The training set can be augmented by slicing these samples. The number of cardiac arrhythmia categories in the training set after slicing is shown in Table 1.

#### Dimension expansion

We added a dimension to the two-dimensional ECG, so that the dimension of the 12-lead signal satisfies the requirements of 2D-convolution (Conv2D) layer for the dimension of the input data. We call the newly added dimension the channel dimension. The two-dimensional ECG $A \in \mathbb {R}^{8192 \times 12}$ was expanded into $A \in \mathbb {R}^{8192\times 12\times 1}$, where the length is 8192, the lead number is 12 and the channel number is 1. During the training process, channel number of the output feature map of each convolutional layer changes synchronously with the number of convolution kernels. Figure 1 shows the process of slicing, concatenating and expanding dimension of the original 12-lead ECG.

### DSE-ResNet

Abnormal ECG signals are mainly manifested as changes in waveform shape and periodic rhythm^34^. Some abnormal ECG signals are periodic and appear in almost every waveform cycle, other abnormal ECG signals are sporadic and only occur in a few heartbeat cycle. Meanwhile, patients of different ages and genders may have different ECG signals for same cardiac arrhythmia. Therefore, DSE-ResNet contains ResNet for extracting the internal and inter-lead features and DSE for extracting global features of two-dimensional ECG. Furthermore, we introduce the age and gender as auxiliary features for training.

**Figure 2:**
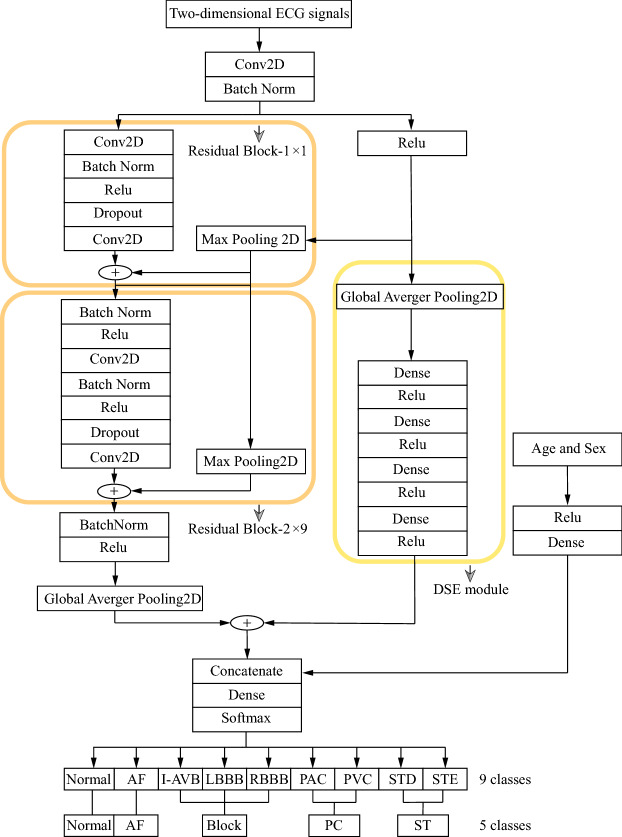
Structure of DSE-ResNet.

Figure 2 shows the overall structure of DSE-ResNet. Residual blocks are commonly used in CNNs to improve gradient flow through the networks and enable training of deeper networks. The ResNet in our model is composed of 1 residual block-1 and 9 residual block-2. Every residual block has 2 Conv2D layers for extracting two-dimensional ECG local features (internal and inter-lead features). The entire residual block has 20 Conv2D layers, where the size of the convolution kernel is (32, 1). The first and last 4 Conv2D layers have 12 and 192 convolution kernels respectively, and the number of convolution kernels is doubled for every 4 Conv2D layers in between. Activate Relu is used to increase the non-linear ability of the model. Batch Normalization and Dropout^35^ play a good role in improving the training speed and preventing overfitting. Shortcut connection is used to complete the identity mapping of features and prevent the phenomenon of gradient disappearance and explosion. The 2D maximum pooling layer in each shortcut connection is used to adjust the dimension of features.

**Figure 3:**
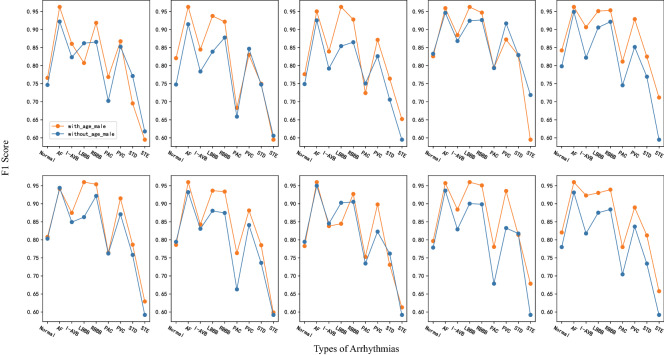
Compare the $F_1$ scores of the models with and without age and gender. The 10 subgraphs used models with different hyper-parameter combinations. Table 2 shows these hyper-parameter combinations.

Squeeze-and-excitation (SE) module^36,37^ can squeeze features in the channel dimension and excite features to a higher-dimensional feature space, which has a global receptive field in a sense. The Detached SE (DSE) module in our model is independent of any residual block. It uses a 2D global average pooling layer to extract global features for each lead of the two-dimensional ECG from the channel dimension. Then the 4 dense layers in the DSE module map the extracted global features to a new feature space. Although the addition of the DSE module will increase the computational complexity of the entire model, it can increase the nonlinearity of the DSE-ResNet and establishes the correlation between channels. Patients with different age and gender may show different waveform states on the same type of cardiac arrhythmia. Figure 3 shows that the $F_1$ scores of cardiac arrhythmias obtained by the multi-group model when age and gender are included are better than those without. Therefore, age and gender are introduced into training as auxiliary features, which is helpful for the DSE-ResNet to capture the influence of basic information of the patient on cardiac arrhythmias.

### Orthogonal experiment

Appropriate hyper-parameters can improve the performance and effect of model learning. We used Orthogonal Experimental Design (OED) to select combination of hyper-parameter values.

OED is a design method for studying multi-factor and multi-level problems. It selects some representative points with uniform dispersion and neatness characteristics from the entire test point for testing based on orthogonality. The process of selecting representative points is often realized by constructing an orthogonal table. Based on relevant research experience, we selected the hyper-parameters that need to be adjusted and gave a corresponding set of estimated values. These estimated values constitute the entire test point of the orthogonal table. We used pairwise independent combinatorial testing (PICT)^38^ to construct an orthogonal table for the selected hyper-parameter values to obtain a representative combination of multiple sets of hyper-parameters. Unlike random selection and grid search, PICT is a selection combination parameter technique used in the field of software testing to reduce the number of system test case inputs. The choice of a large number of hyper-parameters in neural networks is the application scenario of choice for PICT.

### Ensemble model

Ensemble model accomplishes learning tasks by constructing and combining multiple learners^39^. Compared with the classification performance of a single model, ensemble model can often achieve better classification performance and generalization ability^40^. We use ensemble model to reduce the overall error of our model.

The ensemble model contains multiple learners, and each learner is the optimal DSE-ResNet trained based on a representative combination of hyper-parameters. A individual learner is called the single optimal model in this paper. The ensemble model uses a voting strategy to integrate all single optimal models. Specifically, each single optimal model will give a prediction value for the same test sample. Based on the multi-model voting strategy that the minority obeys the majority, the ensemble model takes the predicted value with the most votes as the final output value. Although the use of ensemble model increases the computational complexity, it can effectively improve the classification performance and fault tolerance of the model.

### Ethics statement and consent to participate

The database used in the study was an open access database, https://doi.org/10.1166/jmihi.2018.2442. It can be obtained in https://physionet.org/content/challenge-2020/1.0.1/ or http://2018.icbeb.org/Challenge.html. Therefore, no ethics statement and informed consent is required for this study. All methods in this study were carried out in accordance with relevant guidelines and regulations. This study was carried out in compliance with the Declaration of Helsinki.

## Experimental details

### Software and hardware environment

The proposed model is built and trained using the Keras framework. All experiments are run on a server with Quadro P2200 video card and 5G video memory.

### Data pre-processing

#### Denoising

**Figure 4:**
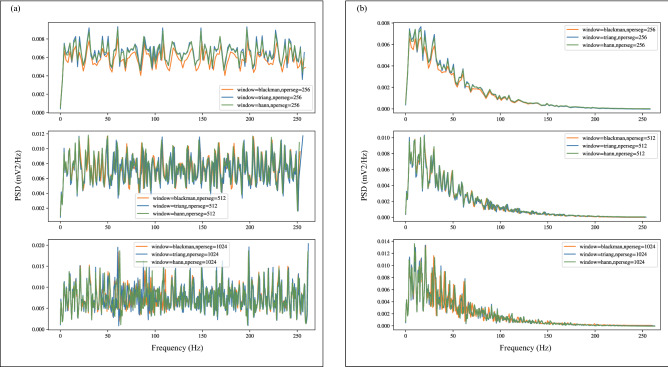
Power spectral density curves obtained by applying Welch with different windows and different window lengths. Windows include Blackman window, Hanning window, and Triangular window. And window lengths include 256, 512, and 1024. (**a**) Power spectral density curve of the lead I signal of the abnormal sample before filtering. (**b**) Power spectral density curve of the lead I signal of the abnormal sample after filtering. Each subplot uses the same window length and a different window.

Muscle noise, power-line noise and baseline wander present in the different ECG leads were removed with a bandpass filter with cutoff frequencies of 0.5 Hz to 49 Hz. Figure 4 shows the power spectral density estimates calculated with the Welch^41^ method for lead I of abnormal sample before and after filtering with the Butterworth bandpass filter^42^. Visualizing the power spectral density curves after applying the welch method with different windows and different window lengths, it is observed that high-frequency noises are attenuated.

**Figure 5:**
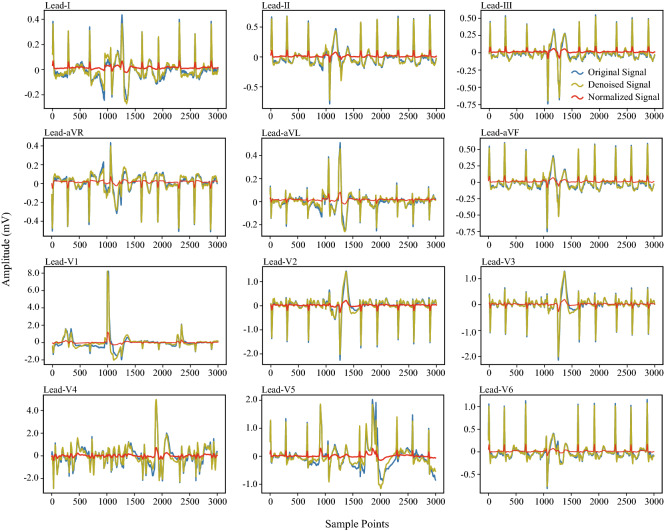
Data pre-processing.

The results of the waveform of A1001 before and after pre-processing are shown in Fig. 5.

#### Min–max normalization

Time series data can take a wide range of values in some cases, so it needs to be scaled to a fixed value interval to speed up the learning process^43^.

The amplitude of the voltage value in the original 12-lead ECG is $[ - 20.9\;{\text{mV}},\;20.7\;{\text{mV}}]$, and the amplitude difference between leads is large. It can be seen from Fig. 5 that the maximum and minimum amplitudes of the original 12-lead ECG are distributed in a symmetrical interval. Therefore, we use Min-Max Normalization^44^ to scale the amplitude of the voltage value of the two-dimensional ECG to the symmetrical interval $[ - 3\;{\text{mV}},\;3\;{\text{mV}}]$, which is

2 $$\begin{aligned} A_{ij} = R_\mathrm{min} + \frac{(R_\mathrm{max}-R_\mathrm{min})(A_{ij}-A_\mathrm{min})}{A_\mathrm{max}-A_\mathrm{min}}, \end{aligned}$$

where $R_\mathrm{max} = 3\mathrm{mV}$ and ${R_\mathrm{min}} = -3\mathrm{mV}$ represent the boundary value of the normalized interval, $A_{ij}$ is the voltage value in the *i*-th row and *j*th column of the two-dimensional ECG, $A_\mathrm{max}$ and $A_\mathrm{min}$ respectively represent the maximum and minimum voltage value in the two-dimensional ECG. Figure 5 shows the normalized result.

### Choice of hyper-parameters

We use OED to determine the combination of hyper-parameters values. Firstly, the batch-size is controlled to the maximum limit that the experimental machine can withstand. Secondly, three hyper-parameters are selected for orthogonal experiment, including learning rate, dropout and momentum. According to the experience of the previous experiments, the value set of learning rate is [0.05, 0.1, 0.15], the value set of dropout is [0.3, 0.5, 0.8], and the value set of momentum is [0.5, 0.7, 0.9]. We use PICT to construct an orthogonal table to combine and match preset values. Table 2 shows the combination of preset values of hyper-parameters configured through PICT. Five-fold cross-validation is used for the models for each set of hyper-parameter combinations, and then the one-fold model with the lowest average loss in the validation set is selected as the single optimal model.

**Table 2:** Orthogonal table of hyper-parameters.

No.	Learning rate	Dropout	Momentum
1	0.1	0.8	0.5
2	0.15	0.3	0.9
3	0.05	0.5	0.5
4	0.15	0.8	0.7
5	0.05	0.3	0.7
6	0.1	0.5	0.9
7	0.15	0.3	0.5
8	0.05	0.8	0.9
9	0.1	0.3	0.7
10	0.15	0.5	0.7

## Results

### Performance metric

The classification performance of the algorithm can be evaluated by accuracy, precision, specificity, sensitivity, and $F_1$ score^45,46^. For multi-classification tasks, the average $F_1$ score^47^ is an important indicator to measure classification performance. The $F_{1i}$ score of the *i*th cardiac arrhythmia is the harmonic average of precision $F_\mathrm{P}$ and recall $F_\mathrm{R}$, where $F_\mathrm{P}$ describes how many of the predicted positive samples are true positive samples, $F_\mathrm{R}$ describes how many true positive samples are picked out. Specifically, the $F_{1i}$ score is defined as:

3 $$\begin{aligned} F_{1i} = \frac{2(F_\mathrm{P} \times F_\mathrm{R})}{F_\mathrm{P} + F_\mathrm{R}}, \end{aligned}$$

where $F_\mathrm{P}= \mathrm{TP}/(\mathrm{TP + \mathrm FP})$ and $F_\mathrm{R}= \mathrm{TP}/(\mathrm{TP + \mathrm FN})$, TP is the number of positive samples that are classified to be positive, FP is the number of negative samples that are classified to be positive, and FN is the number of positive samples that are classified to be negative. The average $F_1$ score among types is a comprehensive evaluation indicator for evaluating the overall performance of the model, which is defined as:

4 $$\begin{aligned} F_{1} = \frac{1}{9}\sum _{i=1}^9 F_{1i}. \end{aligned}$$

We also calculate the $F_1$ scores of 4 sub-abnormal types, i.e., the AF, block, premature contraction (PC) and ST-segment change (ST), where block consists of I-AVB, LBBB and RBBB, PC consists of PAC and PVC, and ST consists of STD and STE. In addition, accuracy, sensitivity, and specificity are also used as performance metric, and they are defined as:

5 $$Acc_{i} = \frac{{{\text{TP + TN}}}}{{{\text{TP + TN + FP + FN}}}},$$

6 $$Se_{i} = \frac{{{\text{TP}}}}{{{\text{TP + FN}}}},$$

7 $$Sp_{i} = \frac{{{\text{TN}}}}{{{\text{TN + FP}}}},$$

where TN is the number of negative samples that are classified to be negative. It should be noted that recall $F_R$ and sensitivity are numerically the same.

### Performance on the small number of test set

We compared the $F_1$ scores of the single optimal models and the ensemble model based on the small number of test sets (500 ECG samples), where each single optimal model is an optimal model trained based on a representative combination of hyper-parameters in Table 2, and the ensemble model is based on the voting strategy to integrate all single optimal models.

Table 3 shows the $F_1$ scores of single optimal models and the ensemble model in the small test set. An important result is that compared to the single optimal models, the ensemble model achieved the highest $F_1$ scores in LBBB, PAC, STE and PC. More importantly, the average $F_1=0.843$ of the ensemble model is greater than that of the single optimal models. The result shows the advantages of the ensemble model compared to the single optimal model, it can effectively improve the fault tolerance of the model and improve the performance of the model classification.

**Table 3:** Comparison of $F_1$ scores between the ensemble model and the single optimal models on the small number of test set.

No.	Average $F_1$	Normal rhythm and 8 cardiac arrhythmias									4 sub-abnormal types			
		Normal	AF	I-AVB	LBBB	RBBB	PAC	PVC	STD	STE	AF	Block	PC	ST
1	0.783	0.739	0.962	0.846	0.786	0.912	0.742	0.854	0.659	0.545	0.962	0.885	0.793	0.629
2	0.816	**0.821**	0.963	0.845	0.938	0.922	0.683	0.830	0.750	0.595	0.963	0.903	0.761	0.709
3	0.810	0.745	0.955	0.821	0.970	0.928	0.682	0.860	0.730	0.595	0.955	0.905	0.773	0.695
4	0.776	0.738	0.933	0.824	0.938	0.915	0.692	0.806	0.742	0.400	0.933	0.896	0.747	0.688
5	0.835	0.787	0.954	0.876	0.938	**0.941**	0.744	**0.907**	0.763	0.606	0.954	**0.926**	0.826	0.728
6	0.824	0.783	0.919	0.851	0.938	0.932	0.738	0.892	0.761	0.600	0.919	0.912	0.814	0.727
7	0.817	0.763	**0.969**	0.830	0.941	0.938	0.736	0.876	0.762	0.541	**0.969**	0.911	0.807	0.704
8	0.780	0.743	0.938	0.804	0.811	0.902	0.710	0.870	0.686	0.556	0.938	0.867	0.789	0.652
9	0.832	0.760	0.938	0.857	0.941	0.931	0.742	0.914	**0.779**	0.629	0.938	0.914	0.824	0.743
10	0.828	0.787	0.942	**0.901**	0.909	0.919	0.742	0.864	0.778	0.606	0.942	0.914	0.802	**0.738**
EM	**0.843**	0.787	0.949	0.870	**0.970**	0.935	**0.764**	0.897	0.748	**0.667**	0.949	0.922	**0.830**	0.729

Significant values are in bold.

### Performance on the CPSC2018 hidden test set

**Figure 6:**
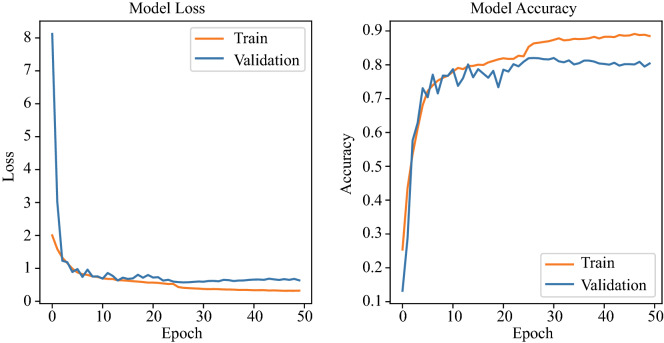
Training/validation set loss and accuracy curve for CPSC2018.

**Figure 7:**
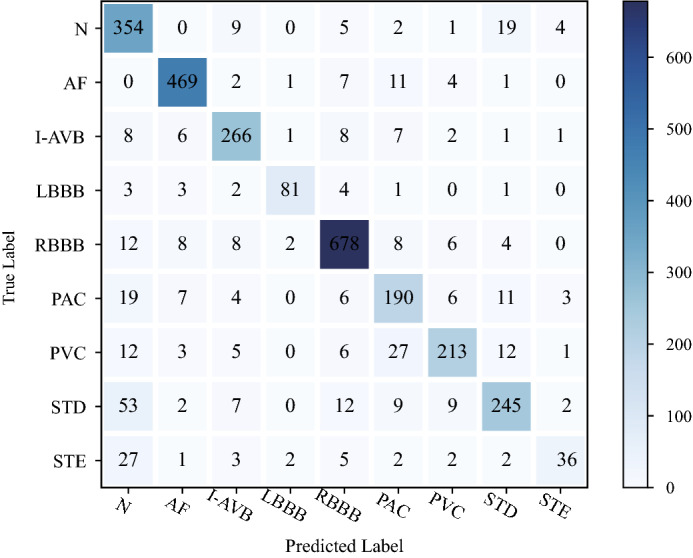
Confusion matrix.

Figure 6 shows the variation of the loss curve and accuracy curve of a single optimal model (Learning rate = 0.15, Dropout = 0.5, Momentum = 0.7) on training set and validation set. The validation set is mainly used to observe how the loss and accuracy curves of the model change during training. According to the performance of the model in the validation set during the training process, it can be judged whether the model is overfitting. The accuracy and loss curve of the model tends to be stable from the 30th epoch in Fig. 6. We have tried increasing the epoch to 70 and found overfitting. Therefore, the method of early stopping is used to reduce the number of training to 50 epochs. By submitting our model to the competition official of CPSC2018, we get the test results based on the hidden test set (2954 ECG records). Figure 7 shows the visual confusion matrix. For the sub-abnormal type ST, 53 samples with STD label and 27 samples with STE label are predicted to be Normal, and 19 samples with the Normal label are predicted to be STD. DSE-ResNet is not sensitive to changes in the ST, which may be due to the scarce number of samples of the ST and the highly similar waveform structure between ST and Normal. Furthermore, doctors disagree on the diagnosis of ST^48^, leading to incorrect labeling of samples, which may also be one of the reasons. For sub-abnormal types AF and Blocks, the proposed model achieved $F_1$ scores of 0.944 and 0.913, respectively.

According to the confusion matrix, we calculated the specific classification performance of DSE-ResNet on the hidden test set. Table 4 shows the accuracy, precision, sensitivity, and specificity of different cardiac arrhythmias. The average accuracy and average specificity of normal rhythm and 8 cardiac arrhythmias are 0.965 and 0.979, and both achieve the maximum value on LBBB, which indicates that DSE-ResNet has high misdiagnosis rate for LBBB recognition.

Table 5 shows the average $F_1$ and the $F_1$ of 4 sub-abnormal types of our model and the top five models with the highest average $F_1$ in CPSC2018. Note that the test results of the models in Table 5 are based on the same hidden test set. Tests show that the proposed model has the average $F_1=0.817$, which is only 0.02 behind the state-of-art model. It is worth noting that the proposed model achieves the best test results in 2 sub-abnormal types, which are $F_\mathrm{AF}=0.944$ and $F_\mathrm{Block}=0.913$, respectively. At the same time, the test results based on the hidden test set show that the model learns internal and inter-lead features from two-dimensional ECG is more sensitive to the ability of AF and Block recognition.

Table 6 compares the classification performance of DSE-ResNet and previous work on the hidden test set of CPSC2018. The results in the table show that the model proposed in this paper achieved $F_1$ scores of 0.944, 0.878, 0.890, and 0.755 in AF, I-AVB, LBBB, and PAC, respectively. The average $F_1$ score is also the highest. Compared with other methods, the simultaneous learning of internal and intra-lead features used in this paper facilitates the identification of multiple types of cardiac arrhythmias.

In summary, compared with the top five models in CPSC2018, DSE-Resnet achieved performance improvement in identifying 2 sub-abnormal types. The average $F_1$ score was also improved compared with most studies, which indicating that DSE-Resnet has certain advantages in detecting some cardiac arrhythmias.

**Table 4:** Other performance metrics of the DSE-ResNet on CPSC2018 hidden test set.

Metrics	Average	Normal rhythm and 8 cardiac arrhythmias									4 sub-abnormal types			
		Normal	AF	I-AVB	LBBB	RBBB	PAC	PVC	STD	STE	AF	Block	PC	ST
$Acc_i$	0.965	0.936	0.978	0.972	**0.992**	0.962	0.954	0.963	0.946	0.979	0.978	0.946	0.943	0.930
$F_p$	0.845	0.725	**0.940**	0.869	0.931	0.927	0.739	0.877	0.828	0.766	0.940	0.912	0.806	0.819
$Se_i$	0.803	0.898	**0.947**	0.887	0.853	0.934	0.772	0.763	0.723	0.450	0.947	0.914	0.768	0.671
$Sp_i$	0.979	0.942	0.986	0.983	**0.998**	0.972	0.972	0.987	0.978	0.996	0.986	0.952	0.970	0.975

Significant values are in bold.

**Table 5:** Comparison of $F_1$ scores with the top five models in CPSC2018.

Model	Average $F_1$	$F_\mathrm{AF}$	$F_\mathrm{Block}$	$F_\mathrm{PC}$	$F_\mathrm{ST}$
Our model	0.817	**0.944**	**0.913**	0.786	0.738
Chen et al.^28^	**0.837**	0.933	0.899	**0.847**	**0.779**
Cai et al.^29^	0.830	0.931	0.912	0.817	0.761
He et al.^49^	0.806	0.914	0.879	0.801	0.742
Yu et al.^29^	0.802	0.918	0.890	0.789	0.718
Yan et al.^29^	0.791	0.924	0.882	0.779	0.709

Significant values are in bold.

**Table 6:** Comparison for classification performance of previous works and ours evaluated on the CPSC2018 hidden test set.

Work	Model	$F_1$ scores									
		Normal	AF	I-AVB	LBBB	RBBB	PAC	PVC	STD	STE	Average
Yao et al.^50^	CNN + LSTM	0.753	0.900	0.809	0.874	0.922	0.638	0.832	0.762	0.462	0.772
Liu et al.^51^	CNN + Expert feature	**0.82**	0.91	0.87	0.87	0.91	0.63	0.82	**0.81**	**0.60**	0.81
Liu et al.^51^	CNN	0.80	0.89	0.87	0.77	0.90	0.65	0.79	0.80	0.56	0.78
Wang et al.^52^	CNN + Attention	0.79	0.93	0.85	0.86	0.93	0.75	0.85	0.80	0.56	0.813
Yao et al.^53^	CNN + LSTM + Attention	0.789	0.920	0.850	0.872	**0.933**	0.736	**0.861**	0.789	0.556	0.812
Our model	CNN + Channel Attention + ensemble model	0.803	**0.944**	**0.878**	**0.890**	0.931	**0.755**	0.816	0.72	0.567	**0.817**

Significant values are in bold.

## Conclusion

In this paper, we propose a general model based on the two-dimensional ECG and DSE-ResNet to realize the automatic classification of normal rhythm and 8 cardiac arrhythmias. The two-dimensional processing method combines the original 12-lead ECG into the same two-dimensional space, so that DSE-ResNet can simultaneously extract the internal and inter-lead features of the 12-lead ECG. Orthogonal experiment instead of grid search to select hyper-parameters reduces the computational complexity. Furthermore, the ensemble learning model based on voting strategy is used to improve classification and generalization performance. Experiments based on the small number of test set show that the classification performance of the ensemble learning model is much better than that of single models. Then we submitted our model to the competition official of CPSC2018 and got the test results based on the hidden test set. The comparison with the results of the top 5 models in the CPSC2018 shows that our model is reasonable in the average $F_1$ value, and achieved the best test results in 2 sub-abnormal types.

This suggests that automatic classification of AF and Block may depend on the relationship between leads. This also means that the use of DSE-ResNet to process multi-channel ECG signals to capture internal lead and inter-lead features is effective for automatic identification of cardiac arrhythmias.

Our results not only provide a new perspective on the automatic classification of cardiac arrhythmia based on the 12-lead ECG, but also raise several questions. Based on the two-dimensional ECG, future research directions include exploring how to further improve the accuracy of prediction, how to reduce the prediction time, how to find redundant leads in the 12-lead ECG, and so on.

## Data Availability

The train datasets used during the current study available in the The China Physiological Signal Challenge 2018, http://2018.icbeb.org/Challenge.html. The test datasets used during the current study are not publicly available for scoring purposes, but test scores can be obtained by submitting the model to The China Physiological Signal Challenge 2018. The datasets generated and analysed during the current study are available from the corresponding author on reasonable request.
